# Prevention of malnutrition among children under 5 years old in Iran: A policy analysis

**DOI:** 10.1371/journal.pone.0213136

**Published:** 2019-03-07

**Authors:** Mohammad Mohseni, Aidin Aryankhesal, Naser Kalantari

**Affiliations:** 1 School of Health Management and Information Sciences, Iran University of Medical Sciences, Tehran, Iran; 2 Health Management and Economics Research Center, Iran University of Medical Sciences, Tehran, Iran; 3 Department of Health Services Management, School of Health Management and Information Sciences, Iran University of Medical Sciences, Tehran, Iran; 4 Department of Community Nutrition, Faculty of Nutrition Sciences and Food Technology, Shahid Beheshti University of Medical Sciences, Tehran, Iran; BITS Pilani, INDIA

## Abstract

**Background:**

Malnutrition is one of the main causes of death in children under 5 years of age and one of the most common factors threatening children’s life and health. Nutrition policy analysis and solving existing problems in children can reduce the effects of malnutrition. This study aimed to analyze the current policies of malnutrition prevention in children under five years of age in Iran.

**Method:**

This study was conducted in 2017 to analyze policies using the "policy triangle framework". In order to examine the policy-making process, the Kingdon’s multiple streams model was used. A combination of two sampling methods, including purposeful and snowball sampling, was applied to select the interviewees. In relation to the implemented documents and policies, the country’s most important policies were selected based on the suggestions of policy makers as well as searching scientific databases and electronic portals. A data collection form was used to identify the current policies and documents and a semi-structured interview guide form was used for the interviews. The framework analysis and MaxQDA software were applied to analyze the data obtained from the interviews.

**Results:**

The key factors affecting policies in Iran included the status of indicators as well as economic, social, structural-legal, policy and international factors. Among the most important policies and implemented programs, the following can be mentioned: growth monitoring, oral rehydration, breastfeeding, immunization, female education, family spacing, food supplementation, nutrition for children under five years of age, and control of nutritional deficiencies. Currently there is a need for a nationwide program and comprehensive document in the field of the nutrition in children under 5 years of age, which requires strengthening of the political process. Participants and stakeholders in nutrition-related policies for children under the age of five were divided into four categories of governmental, semi-governmental, non-governmental, and international organizations.

**Conclusion:**

More attention should be paid to the shortage of some micronutrients, accurate implementation of breastfeeding programs, supplementary nutrition, fortification and supplementation programs for children and mothers, utilization of the advantages of each region and its resources, and better coordination between organizations and their policies, and finally strong incentives are needed to promote macro nutritional goals for children under five years of age.

## Introduction

Malnutrition is a common and widespread condition that usually occurs as a deficiency in the intake of energy, protein, or micronutrients [1]. Malnutrition is one of the main causes of the death of children under the age of 5 years and is one of the most common causes of the decline in the health and life of children, which results in decreased learnability, inefficiency, and inability to acquire skills [2]. Malnutrition contributes to the death of nearly half of the children under five years of age in Asia and Africa. Inadequate nutrition increases the risk of death due to common infections, increases the number and severity of the infections, and may lead to delayed recovery [3–7].

Three important indicators used in estimating malnutrition are stunning (low height for age), wasting (low weight for height), and underweight (low weight for age) [8]. According to a UNICEF report in 2014, the prevalence of underweight, stunning, and wasting in the world was 15%, 25%, and 8%, respectively [9]. The statistics for Iran, according to the latest national study (Demographic and Health Survey, 2010), were 4.08%, 6.83%, and 4%, respectively [10]. In 2012, the World Health Assembly approved a resolution on a comprehensive maternal and child nutrition plan, including six global nutritional goals for 2025. The goals included a 40% reduction of the global number of children under five who are stunted, a 50% reduction of anemia in women of reproductive age, a 30% reduction of low birth weight, no increase in childhood overweight, an increase in the rate of exclusive breastfeeding in the first six months up to at least 50%, and finally, reduction and maintenance of childhood wasting to less than 5% [11, 12]. Worldwide reports on stunting show that the number of children with a short stature during reduced from 255 million to 159 million from 1990 to 2014 [13]. In developing countries, it is estimated that the prevalence of nutritional stunting in children will decrease from 29.8% in 2000 to 16.3% in 2020 [14].

Studies in some countries show that despite economic development, malnutrition in children is still a major health problem in developing countries [15, 16]. A review of the content of nutrition policies in different countries suggests that these policies have the same generalities mostly aiming at supporting children, especially vulnerable children [6]. Regarding the content of food and nutrition policies in Iran, the National Document for Nutrition and Food Security can be considered as the most comprehensive document available among the existing documents. This document has been designed in accordance with the definitions of nutritional security and food security, global experiences, analytical reviews of previous food and nutrition reports and plans, and the views of experts and intersectional stakeholders [17, 18]. The outcomes of policies on prevention of malnutrition in children in Iran indicate their overall success in reducing child malnutrition; however, there are still problems with existing policies and policymaking. Therefore, this study was conducted to analyze the current policies of malnutrition prevention in children under the age of five years in Iran.

## Methods

This study was conducted in 2017 to analyze policies implemented for malnutrition prevention among children under 5 years old in Iran using the "policy triangle framework". This model covers four general areas: context, content, policy-making process, and actors [19]. In the present study, Kingdon’s multiple streams model was used for the policymaking process phase. This model provides a basis to understand the policymaking process as a fluid cycle of stages with the key stages being agenda setting, policy formation, implementation, and evaluation. Moreover, the Kingdon’s multiple streams model was used to examine the agenda setting phase. This model is presented with an emphasis on the agenda, and contains three independent streams as the problem stream, policy stream, and politics stream, which reach each other in a place called "windows of opportunity" and lead to adopting an appropriate policy for problem solving. In other words, the streams come together and open a window of opportunity and then governments decide to address it [20]. Textual review and document analysis were used to identify policies. The main focus at this stage was selection of the main policies of the Ministry of Health and policies that were interlinked by the Ministry of Health. Subsequently, semi-structured interviews were conducted with senior policy makers and nutrition senior managers as key informants. The findings were reported in accordance with COREQ (consolidated criteria for reporting qualitative research) guidelines [21].

### Sampling

A combination of two sampling methods, including purposeful and snowball sampling (expert sampling), [22] was applied to select the interviewees. For interviews, purposeful sampling was used to identify the participants. The samples were selected based on a search of the profession and scientific background in related organizations as well as introduction of previous interviewees [23]. Interviews were conducted until data saturation was reached. Accordingly, twenty-five interviews were conducted with informants and policy makers in this field. Subjects with sufficient knowledge (publication of books, articles, reports, etc.), and sufficient experience in matters related to child nutrition and formulation of policies, plans, and executives were selected to participate in the study. Moreover, willingness to participate in the study was another inclusion criterion. Considering the implemented documents and policies, the country’s most important policies were selected based on search of Internet databases and portals and introduction by policy makers.

### Data collection

For policy documents and state and organizational laws was done in scientific databases and electronic portals. Internet sites related to relevant organizations were also searched in order to access the documents. The official reports published by organizations were also reviewed by visiting the organizations. The documents reviewed included the Constitution of the Islamic Republic of Iran, Iran’s 20-Year Vision Plan, Fourth and Fifth Five-Year Development Plans, Comprehensive Scientific Map of Iran, comprehensive scientific roadmap of the health system, health system reform plan, health indicators in the Islamic Republic of Iran, document of poverty reduction and targeting of subsidies, reports published by the Health and Food Security High Council, and other relevant organizations.

Face-to-face interviews were conducted to collect experts’ opinions at their workplace by a trained experienced male researcher that has experience interview (MM, PhD of health policy). The data collection tool for interviews was also a semi-structured interview guide form. The interview guide was pilot-tested in five interviews and then, based on the results, its draft was modified. A tape recorder was used with the consent of the participants. In addition, notes were taken during the interviews. Data collection was done between January and April 2017. None of the participants was known to the research team prior to participation. During the interview, only the interviewee and the interviewer were present in the room. Each interview took between 30 and 90 minutes. The compilation tool was a data collection form for identifying the current documents and policies in the field of child nutrition and malnutrition prevention.

### Interviews

The main questions of the interview included the following: What is your point of view about nutrition of children under the age of 5 years and the situation in Iran? What has been so far the content of nutrition policies for children under 5 years? What is the process of policy development? What are the underlying factors affecting the development and implementation of malnutrition prevention policies and programs for children under five years? Who are the stakeholders and actors in this field? What are their roles? What are the challenges and solutions of policymaking and implementation of policies? What are your proposed policies and what features should they include? What are the key factors affecting malnutrition in children under the age of five in Iran? To validate this form, the opinions of the supervisors and consultants were applied and the texts related to the topic were reviewed. Transcripts were returned to participants for comment and/ or correction.

### Data analysis

The data from the documents were analyzed using the content analysis technique. The policy triangle model was used in this study for policy analysis. After identifying child nutrition documentation and policies implemented by the Ministry of Health and Medical Education or those implemented in an intersectoral manner by participation of the Ministry of Health, document analysis was carried out. Moreover, framework analysis was used to analyze the data collected from semi-structured interviews to obtain the viewpoints of experts. This method is widely used for research in health systems and policies [24]. The interviews were first transcribed and then coded and grouped (MM and NK). After grouping, there was a logical relationship between the data for which MaxQDA software was used (AA). AA is a male faculty member (PhD) that has previously conducted qualitative studies and had substantial knowledge about working with the software. NK is a pediatrician (MD) and has substantial knowledge of the topic of the present study; therefore, a constant comparison approach [25] was used to avoid confirmation bias. To ensure the reliability of the extracted codes and themes, interobserver reliability (MM and AA) was used and disputes were resolved through discussion. To ensure data rigor, the immersed data were repeatedly referred to and necessary corrections were made in coding and in some cases, re-coding. Peer check was also used by sharing the implemented texts and extracted codes with other colleagues and applying their comments in the results [26].

### Ethical issues

The time and location of the interviews were generally determined by the participants. The objectives of the study were explained to the participants before the interview and informed consent was obtained from all of them. The interviews were confidential. This study was approved by the Ethics Committee of Iran University of Medical Sciences (IR.IUMS.REC1394.9221557201).

## Results

In this section, the results of the interviews, document reviews, and policy analyses are presented based on the policy triangle framework in four areas of context, content, policy-making process, and actors. The characteristics of the key informants who were interviewed are listed in Table 1.

**Table 1:** Characteristics of the key informants in the interview.

Position / Job	Education	Experience in nutrition policies(years)	Number	Position / Job	Education	Experience in nutrition policies(years)	Number
Ministry of Health Consultant	Specialist (Pediatrician)	15	1	Director of IRI Broadcasting	Specialist (Pediatrician)	10–12	2
Director General of the Ministry of Health	Specialist (Pediatrician)	20–25	2	Director of the Relief Committee	Specialist (MD)	15	1
Director General of the Ministry of Health	PhD (Nutritional Sciences)	10	1	Expert of the Ministry of Health	PhD (Nutritional Sciences)	10	1
Director of Ministry of Agricultural Jihad	Master (Agricultural Science)	10	1	Expert of the Ministry of Health	Master (Nutritional Sciences)	8–15	3
Advisor to the Ministry of Industry, Mine and Trade	Master (Industry)	15	1	Expert of the Ministry of Welfare	Master (Sociology)	5	1
Director General of the National Standardization Organization	Master (Food industry)	12	1	Expert of the welfare organization	Master (Midwifery)	8	1
Faculty member of the Institute of Nutrition Research and Food Industry	Specialist (Nutritional Sciences)	10	2	Expert of institute of nutrition research and food industry	Masters (Nutritional Sciences)	8–12	2
Faculty member	PhD (Nutritional Sciences, (Pediatrician, Pediatric nursing)	4–15	4	Director of NGO	PhD (epidemiology of nutrition)	20	1

### Context

Context refers to the social, economic, political, and cultural context and other terms of the policy environment. The basic factors affecting nutrition-related policies for children under the age of five were divided into six main categories (Table 2). This finding was derived from interviews and document analyses.

**Table 2:** Underlying factors affecting nutritional policies for children under 5 years of age in Iran.

main factors	Subordinate factors
Status of indicators	Demographic, nutrition, health, education indicators, related to women’s economy
Economic factors	GDP, poverty, inflation rate, annual income rate, economic participation of active population
Social factors	Traditions, culture, lifestyle, health services, parental education, maternal employment, household population, media
Structural—legal	Organizational structure, interconnections, management, rules and regulations
Policy	Policies, nutritional policy decisions, evidence-based policymaking
International	International policies, relations with international organizations

One of the key interviewees said, "Apart from the economic factor, there is a cultural factor. The main factors in our country are ignorance, and inappropriate nutritional behaviors. Awareness, nutrition literacy, and public health are important. Inappropriate nutritional behaviors, such as increased consumption of fast foods, usually results from low nutrition literacy and a poor consumer culture. Moreover, individual and social responsibility are important factors." Participant 2.

Another interviewee said, "especially in nutrition, media policies are very important, such as promotion of harmful substances and junk food such as potato chips, or on the contrary, promotion of milk consumption, economic policies, job creation, welfare, fair income distribution, social justice, all of them are effective. However, the economic status of the family is associated with the amount of food, and food consumption also affects the nutritional status of children" P 5.

Among the main factors, indicators are of great importance. Demographic indicators are indicators such as birth rate, mortality rate, and life expectancy. Table 3 shows the status of some of these indicators based on a UNICEF report in 2016 [27].

**Table 3:** Demographic, health, and economic indicators related to nutrition in children under 5 years.

**Indicators related to nutrition**				**Demographic indicators status**		
Low birth weight (2009–2013)			8%	Iran Population (2015)		79.109.272
Early breast feeding (2010–2010)			69%	Number of children under 5 years old(2015)		6,855,000
Comprehensive breastfeeding at first 6 months (2010–2015)			1990	Annual birthdays		1.350.000
Start feeding with solid foods and soft in 8-6months (201 5–20 10)			2015	Life expectancy at birth (years)		76
Breastfeeding up to 2 years (2010–2015)			51%	Total fertility rate (2015)		1.7%
**Health indicators**				Infant mortality rate (under 1 year) (2015)*		13
Using proper drinking water sources (2015)			96%	Infant mortality rate (2015)*		10
Using proper sanitation facilities (2015)			90%	Mortality rate under 5 years (rank)		104
Caring for children with pneumonia (2010–2015)			76%	Annual population growth rate (1990–2015)		1.4%
Treatment with the ORS in cases of diarrhea (2010–2015)			61%	Crude Birth Rate (2015)		17%
Vaccination coverage	BCG	99%		Raw birth rate	1970	42%
	Polio3	99%			1990	33%
	MCV1	99%			2015	17
**Economic indicators**				**Indicators related to women**		
Gross national income (GNI) per capita (2014)			$ 16.590	Prevalence of contraception (2010–2015)		77%
Average annual inflation rate (1990–2014)			21%	Life expectancy: women against men(2015)		103%
Payments as a percentage of gross domestic product (health)			3%	Adult literacy rate: females against males(2009–2014)		88%
Annual growth rate of GDP		1970–1990	4.3%	Prenatal care (2010–2015)	At least once	97%
		1990–2014	2.4%		At least 4 times	94%

*Rates are per thousand.

One of the interviewees commented on the impact of the economic situation on nutrition, "The statistics show that there is a negative relationship between the socioeconomic status and the body mass; in other words, people of lower economic levels usually have less protein intake and consume more carbohydrates. All of these factors can prevent normal nutrition in children and therefore children might not grow properly …." P 2.

Another important factor affecting the economic state of the family was the individual’s income or, in other words, occupation, "You should not forget unemployment. An unemployed person faces many problems. Its first effect can be on nutrition. Generally, wasting and underweight are more visible in families with lower incomes." P 10.

Another factor that had a significant impact on household consumption was the price of food and inflation. Most interviewees believed that high growth of prices had a very negative effect on food consumption, "One of the main causes of decreased consumption of some foods is their price. Pricing policies are important. Changes in food prices, especially basic goods, particularly when the revenue growth is not proportional to the rising prices, have a large impact on the consumers’ behavior and reduce people’s access, especially vulnerable groups, to food products, because as soon as the price rises, people will quickly take them out of their food basket" P 8.

### Content

Government policies and programs designed to promote health need to consider provision of healthy, appropriate, affordable food products for the family head and households. The foundation of the policies of most countries is based on global organizations’ policies. In 1983, UNICEF began negotiations to launch a child survival plan and a development revolution for children. In this case, GOBI-FFF can be referred to as one of the most important UNICEF global policies for child health. This term stands for seven programs, i.e. Growth monitoring, Oral rehydration, Breastfeeding, Immunization, Female education, Family spacing, and Food supplementation, as a selective primary health care strategy [28]. The program was also adopted in Iran and the plans were implemented accordingly.

Studies on the content of the policies adopted in Iran have shown that the main policies related to child nutrition are divided into two main categories: policies that are consistent with the GOBI-FFF and policies with focus on the quality of life. Nutrition promotion and improvement policies can be referred to as the most important policies in this group. These polices include three main policies as breastfeeding, nutrition of children under 5 years, and control of micro-nutritional deficiencies, including iron, iodine, vitamin A, and vitamin D.

Although several plans were made in the past years to develop a nutrition-related document in the Ministry of Health and even some documents were developed at a national level, some weaknesses in the execution of the strategies proposed in the documents has hindered the expected progress. In this regard, a national nutrition and food security document was developed aiming at supplying a national program for promoting nutrition and food security. This document is considered a comprehensive document and reference for nutrition and food security [17]. At present, a comprehensive national nutrition document is required for children under 5 years.

### Policymaking process

The policymaking process consists of four parts: identification of the problem and the agenda, policy formulation, policy implementation, and policy evaluation. The findings of the nutrition policymaking process for children under 5 years in Iran, based on interviews and documents, are presented in Table 4.

**Table 4:** Summary of the findings of the nutrition policymaking process for children under 5 years.

Policy-making process	Components	Participants’ direct quotes
**Reasons to be on the agenda**	Top documents emphasizing food and nutrition security	"Our upstream documents have always protected mother and child matters and consider it necessary to care for them." P 3
	Special viewpoints of policymakers on the issue of mother and child	"Policymakers, at least within the past 30 years, have focused on mother and child matters and have paid more attention to them compared to other groups." P 2
	High prevalence of malnutrition in children under 5 in poor areas of country	“By dividing the provinces of our country, we know that in deprived areas, the extent of the problem is much worse than the average of the country." P 4
	Important role of nutrition in the first years of life, particularly the first 1000 days and the first 5 years of life	"The role of nutrition and food status is unique in children under five years and within the first one thousand days of life, which is not comparable with other ages at all." P 1
	Results of the data from national studies on the presence of a serious and fundamental problem	"These are the data that have created the bases, developed the main questions stating that the health system should solve the basic problems or the questions it has raised." P 12
	Food and nutrition insecurity in some parts of country	"There are also some who are poor, 20–23% of the households do not receive enough energy, i.e. they are insecure." P 4
	Deficiency of some micronutrient in country	"Now we have indicators that show there are a lot of problems in our country in terms of micronutrients." P 16
	Pressure from stakeholders	"There is always pressure from stakeholders, official organizations like Committee of Relief and Welfare and unofficial child supporters like NGOs, as well as mothers, media, people, etc." P 2
	Feedback from other organizations	"Finally, we also receive feedback from other organizations, for example, a supporting organization such as the Relief Committee says many children have nutritional needs." P 10
	need for promotion of indicators in accord with international policies	"WHO believes that if you reduce malnutrition to below 5% in a country, you have reached the indicators you want. Just work on upgrading." P 1
	Experiences and actions of other countries	"In addition, the experiences and documents provided and shared by other countries led to adopting a policy to eradicate malnutrition." P 14
**Policy formulation**	investigating existing status	"Our work is based on the fact that we assess the current situation of the most common problems in the country to find common nutritional problems in the country." P 9
	Performing assessment studies	"This information is evaluated using the data provided in the status check." P 13
	Prioritizing problems	"When the initial problems are known, we will prioritize them according to their importance." P 17
	Involving different sectors and organizations	"In order for a proper and accurate policy to be elaborated, we must use all organizations that will be involved in implementation later." P 3
	Adopting a proper policy	"Effective interactions and adoption of an intervention based on specific national circumstances and cost-benefit and cost-effectiveness analysis…” P 1
	Performing small pilot studies	“It should be piloted in one or more cities and then implemented the whole country since we first want to make sure of its effectiveness." P 4
	Doing pilot studies more broadly	"After the initial pilot, we will run a broader pilot, for example, in several different provinces." P 4
	Evaluation of adopted policy	"When these steps are over, we will evaluate their results." P 4
	Preparing a national instruction	“In order to expand it as a national instruction to be implemented across the country…" P 4
**Policy implementation**	From top to bottom	"Thus, a policy must be applied for implementation from somewhere; that is why policies are from top to bottom…. " P 8
	Individual and community-based programs	"Two types of policies are being formulated, one series is individual and the other is community-based." P 5
	Delegation at provincial levels to adapt conditions for policies	"Because of some differences between provinces, general polices are communicated; however, for implementation, some assignments are given to match the conditions." P 15
**Policy evaluation**	Self-evaluation of the activities of each organization	"Due to the extent of the activities of the organizations involved, their programs are evaluated by themselves; it’s right…" P 7
	Need for final evaluation and review of achievements of policy goals by Ministry of Health	"High Council of Health and Food Security should monitor this stewardship. It claims that its stewardship is in the Ministry of Health.” P 1
	Monitoring while implementing policies	"Programs and policies are monitored as they are being implemented.” P 18

**Fig 1:**
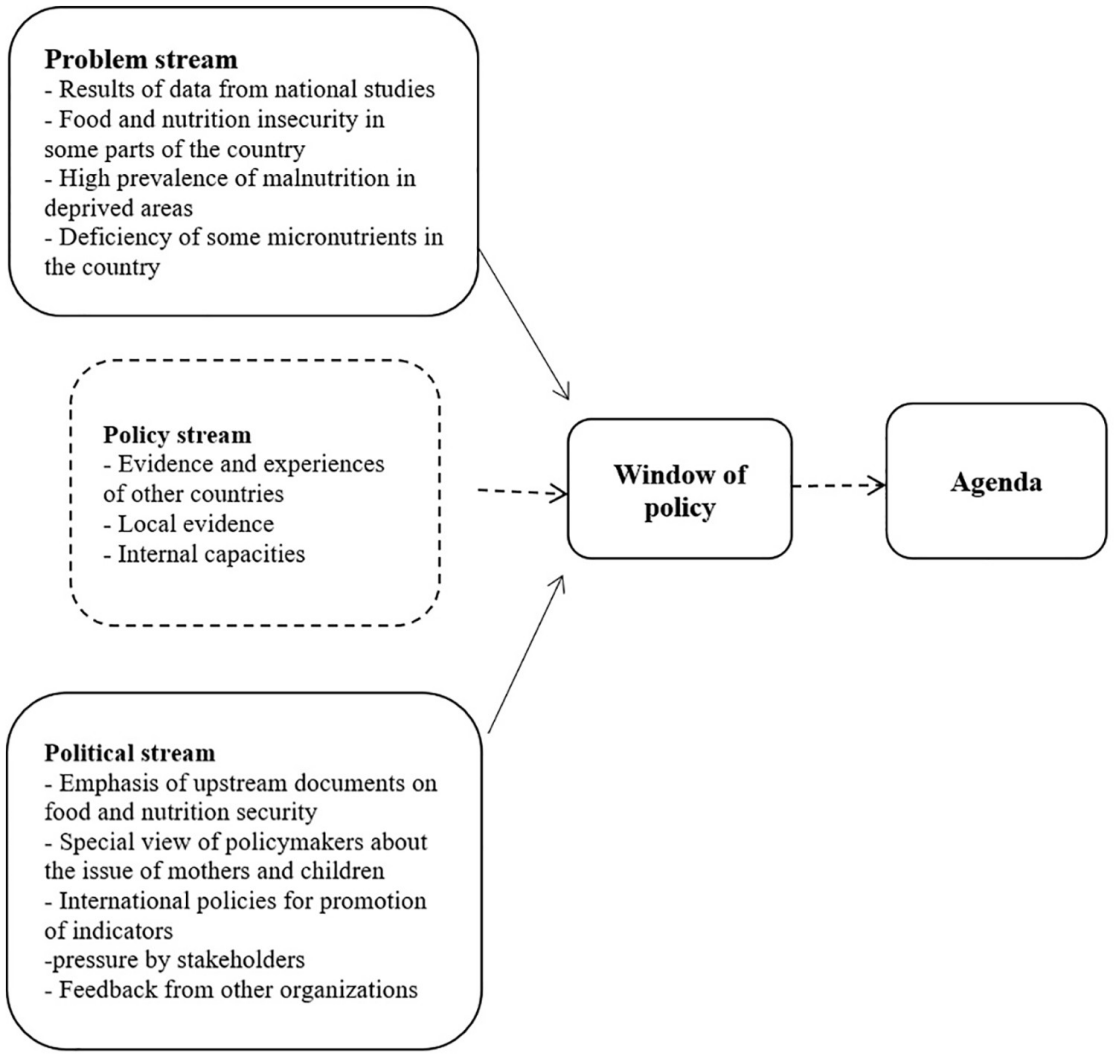
Multiple streams in the process of nutrition policies agenda for children under five years’ old. According to the Kingdon’s theory, the window of opportunity opens when the three streams exist simultaneously. At present, there is a need to strengthen the political stream so that the window of opportunity can be formed correctly, which seems to be the reason for the lack of a nationwide program and document on child nutrition.

Table 4 provides a review of the documents and interviews related to the policymaking process of nutrition and its stages. The policy-making process is organized in a systematic and scientific manner. Of course not all these steps may be followed in the manner shown in the table. The findings from the agenda are based on the Kingdan’s theory as displayed in Fig 1. Due to the weakness of some currents at different times, the window of opportunity has not been open at all times. Currently children’s nutritional problems are usually overcome based on a national nutrition documents mostly embedded in these policies.

### Actors

It is not usually possible to identify all stakeholders in a problem, but the main stakeholders involved in making informed decisions should be identified. Some organizations play a more significant role because of a closer relationship with child nutrition. Stakeholders and key actors involved in nutrition and food security issues for children under five years are listed in Table 5. This table results from the findings of the interview and document analysis.

**Table 5:** Actors and stakeholders in nutrition policies for children under the age of five.

Organization / government agency	Semi-governmental organization/ agency	Non-governmental organization/agency	International Organization / Agency
Ministry of Health Ministry of Co-operation, Labor and Social Welfare Ministry of Agriculture Ministry of Industry, Mine and Trade Ministry of Education Ministry of Culture and Islamic Guidance Ministry of Research Science and Technology Ministry of Interior Islamic Consultative Assembly Targeting Subsidies Organization Welfare Organization Planning and Budget Organization National Standards Organization Literacy Movement	Islamic Republic of Iran Broadcasting Imam Khomeini Relief Committee	NGOs (Saman) Iranian Children’s Nutrition Science Association Iranian Nutrition Forum Scientific Society of Food and Nutrition Supporter of Health Experts Researchers University professors	The World Health Organization UNICEF Food and Agriculture Organization World Food Program Office

The position of each major stakeholder affecting the nutritional policies of the country is different. The Supreme Council of Health, the Ministry of Health, and the Ministry of Agricultural Jihad are considered as effective stakeholders that highly support the policies. The level of activity and participation of stakeholders and active actors is not the same. Some organizations are more active and some have a lower level. A large number of stakeholders said:

"Because all sectors need to be involved for this purpose, which requires a high level of support from above. If it is to be done at the national level, it should receive support from the Supreme Council of Health and Food Security. The Council has -and should have- a significant role

P 1.

"…Some organizations have the greatest impact on nutrition including child nutrition. The role of the Ministry of Agricultural Jihad is very significant because they are the main food producers in that area. Business is very important, because of supply and demand in the region, because of import and export. Generally we are talking about food security."

P 15.

Among organizations mentioned above, the activity and participation of low-level organizations should be increased considerably. Similarly, organizations such as Islamic Republic of Iran Broadcasting have appropriate activities that should be enhanced because of the importance of these organizations and their effective in nutrition. One of the participants said, "Radio and TV have a very important role in enhancing culture, education, and information. If the public consumption culture is reformed, if their nutritional literacy increases, then, of course, the nutritional status of the whole society and nutrition of our children will improve. A mother who knows how to deal with her child’s nutritional problems, for example, in her illnesses, who knows to take supplements, her child is less likely to suffer from malnutrition." P 3.

As stated above, increasing the participation of organizations in policymaking will lead to increased commitment to policies in the upstream institutions. Lack of participation of stakeholders and actors can lead to lack of proper program execution and failure in the predicted goals, "Experience has shown that if we do not engage all the stakeholders from the very beginning and design a program without their contribution, we definitely will get into trouble during implementation. That is the reason why we ask all of them to be aware of the program schedule; for example, if we are working with children, we ask them to participate in child health management." P 20.

## Discussion

An examination of the policies adopted in Iran indicates that there are adequate nutrition policies for food and nutrition security, but there is no specific document and policy related to nutrition and prevention of malnutrition in children under 5 years although nutrition is very important in this age group.

The findings indicated that the main factors affecting the nutritional status of children were the economic status and poverty of the household. As a result, with improvement of the economic status and the reduction of poverty, the odds of a better nutrition status at higher income levels (e.g. per capita income or monthly income) will increase. Studies in some countries across the world have shown a relationship between income and stunting [29–31]. Paying subsidies to poor people is one of the social support methods that helps improve child nutrition and reduce their malnutrition. Sometimes this contribution can be made conditionally, for example, if the person attends the school or completes the immunization program [32–34].

As for the content of nutrition policies, it can be stated that they should take into account the differences in economic and cultural situations in different parts of the country. Proper implementation of the upstream rules requires attention to the conditions of each region. Policies should be implemented in such a way that, in addition to meeting the objectives included in the relevant laws, they could respond to the different needs of each region. Moreover, the policies that are being adopted, in addition to promoting indicators in low-income areas, should focus on deprived areas with a higher percentage of malnutrition. The content of the policies should address the existing differences and comprehensive growth in all aspects, particularly factors causing differences in the health and nutrition status of children. Every child has the right to have fair opportunities, and each community has a share in expanding these opportunities so that nobody could be deprived of them [30].

Special attention to international goals, plans and resolutions to improve equity in access economic justice can reduce the gap in the prevalence of malnutrition between different provinces of the country. In the definition of food security, the three categories of food availability, food accessibility, and food sustainability are the main parameters. It is expected that establishment of the proposed National Nutrition and Food Security Interventions in the coming years will improve key indicators of nutrition and food security. For instance, all provinces of the country will be ranged from safe to very secure in terms of food security [18].

The review of the content of the policy of other countries also suggests that the policies of different countries vary according to the context and conditions of each country, but similar generalities are found in all of these policies, which are mostly related to protecting children, especially vulnerable children or those at risk. Some nutritional policies of our country have not been able to respond to nutritional problems [35]. To eliminate such cases, healthcare providers need to be aware of the interrelated and complex relationships between demographic characteristics, health status, and health needs of the community [36–38].

The content of the policies should also consider the process of nutritional changes in the society. The current trend in Iranian children is towards the reduction of some micronutrients with an increase in the prevalence of overweight and obesity. One of the solutions is to use the experience of other countries in dealing with nutritional problems. For example, the focus of Australian policies is mainly on ​​food production, obesity and overweight and the Sri Lanka policies mainly focus on promoting community nutrition and reducing malnutrition, networking, changing people’s behavioral and nutritional patterns, and interventions in vulnerable groups [39]. Nutrition policies in the United Kingdom have mainly concentrated on cardiovascular diseases and cancers. To prevent these diseases, attention has been paid to their risk factors such as nutrition and obesity [40].

In the process of child nutrition policymaking in Iran, an important point, which has many positive and negative effects, is management changes and their effects on policy changes. The interests and intentions of political leaders have a direct impact on nutrition policies [39]. It should be noted that nutrition programs and policies are based on upstream documents and management change may cause contradictions between the policies and documents. As for stakeholders, it should be kept in mind that child nutrition requires a strong intersectoral cooperation and custodianship, with the task of providing responsiveness to other organizations involved. A small number of stakeholders believed that custodianship was not required. In a study of nutrition stakeholders in Guatemala, it was emphasized that a consensus among actors in nutrition policies requires comprehensive planning and numerous activities, and countries must design suitable processes for stakeholders’ approval [41].

## Conclusions

The field of child nutrition is very important, but some related issues are not on the policymakers’ agenda as required. Issues such as lack of some micronutrients, which is highly prevalent in children, require policies that need to be pursued more seriously. Precise formulation and implementation of breastfeeding programs, complementary nutrition, and therapeutic supplementation for children, and fortification and sumpplementation for mothers should receive priority in this regard. The advantages and capabilities of each region are among the most important elements to be considered in policies. The use of ethnic and local foods in each region, correction of malnutrition, and promotion of nutritional literacy should be considered in different areas. Furthermore, given the fact that the policies of different organizations affect each other and lack of coordination between these policies may affect the success of the overall policies adopted, there is a strong need for more cooperation to materialize macro objectives.
